# Modulation of benzylisoquinoline alkaloid biosynthesis by heterologous expression of CjWRKY1 in *Eschscholzia californica* cells

**DOI:** 10.1371/journal.pone.0186953

**Published:** 2017-10-27

**Authors:** Yasuyuki Yamada, Tomoe Shimada, Yukiya Motomura, Fumihiko Sato

**Affiliations:** Department of Plant Gene and Totipotency, Division of Integrated Life Science, Graduate School of Biostudies, Kyoto University, Kyoto, Japan; Dokuz Eylul Universitesi, TURKEY

## Abstract

Transcription factors control many processes in plants and have high potentials to manipulate specialized metabolic pathways. Transcriptional regulation of the biosynthesis of monoterpenoid indole alkaloids (MIAs), nicotine alkaloids, and benzylisoquinoline alkaloids (BIAs) has been characterized using *Catharanthus roseus*, *Nicotiana* and *Coptis* plants. However, metabolic engineering in which specific transcription factors are used in alkaloid biosynthesis is limited. In this study, we characterized the effects of ectopic expression of CjWRKY1, which is a transcriptional activator with many targets in BIA biosynthesis in *Coptis japonica* (Ranunculaceae) and *Eschscholzia californica* (California poppy, Papaveraceae). Heterologous expression of CjWRKY1 in cultured California poppy cells induced increases in transcripts of several genes encoding BIA biosynthetic enzymes. Metabolite analyses indicated that the overexpression of the *CjWRKY1* gene also induced increases in the accumulation of BIAs such as sanguinarine, chelerythrine, chelirubine, protopine, allocryptopine, and 10-hydroxychelerythrine in the culture medium. Previous characterization of EcbHLH1 and current results indicated that both transcription factors, WRKY1 and bHLH1, are substantially involved in the regulation of BIA biosynthesis. We discuss the function of CjWRKY1 in *E*. *californica* cells and its potential for metabolic engineering in BIA biosynthesis.

## Introduction

Plants produce structurally divergent, low molecular weight specialized secondary metabolites. Alkaloids, which are nitrogen-containing compounds, are found in approximately 20% of plant species and often used as important pharmaceuticals, stimulants, and narcotics because of their strong biological activities [1]. Despite their usefulness, information on the biosynthetic pathways of alkaloids is very limited to specific plant species. The biosynthesis of monoterpenoid indole alkaloids in *Catharanthus roseus*, nicotine alkaloids in *Nicotiana* plants, and benzylisoquinoline alkaloids (BIAs) in *Papaver somniferum*, *Coptis japonica*, and *Eschscholzia californica*, are well understood at the molecular level [2–5].

Here, we focus on BIAs, because they are among the most diverse alkaloids, with approximately 2500 natural product structures [6]. BIAs include many pharmaceutically valuable chemicals such as the analgesics morphine and codeine (in opium poppy, *P*. *somniferum*), anti-adipogenics, the antimicrobial agent berberine in goldthread (*C*. *japonica*), and the antimicrobial agent sanguinarine in California poppy (*E*. *californica*) [1, 3, 7]. Furthermore, the biosynthetic pathways of these BIAs have been intensively investigated at the molecular level [3, 8]. Several transcription factors have also been identified and characterized, including CjbHLH1 and EcbHLH1-1/EcbHLH1-2 (the homologs of CjbHLH1) from *C*. *japonica* and *E*. *californica*, respectively [9, 10], CjWRKY1 from *C*. *japonica* [11], and PsWRKY from opium poppy [12]. However, metabolic engineering approaches that use transcription factors in BIA biosynthesis are very limited, except for early attempts to use *Arabidopsis thaliana* WRKY1 (AtWRKY1) in California poppy and opium poppy to increase biosynthesis and production of BIAs [13].

In this report, we examined the effects of native WRKY in BIA biosynthesis. WRKYs are some of the most important plant-specific regulators in biotic and abiotic stress responses, development, and senescence [14–16]. The WRKYs contain one or two copies of the WRKY DNA-binding domain, which is composed of approximately 60 amino acids and includes the highly conserved N-terminal motif WRKYGQK and a C-terminal zinc finger motif. CjWRKY1 isolated from *C*. *japonica* cells [11] belongs to Group IIc, a different clade from that of AtWRKY1, which belongs to Group I. However, whereas CjWRKY1 directly binds to the W-box element (TTGACC/T) [17] and functions as a comprehensive activator in BIA biosynthesis in *C*. *japonica* cells, the general function in the biosynthesis of BIAs in other species has yet to be determined. In fact, PsWRKY is proposed to be a transcriptional activator of BIA biosynthesis in opium poppy, because of the binding of PsWRKY to the W-box and the transactivation activity of PsWRKY against the tyrosine decarboxylase gene [12]. However, the actual role of PsWRKY in the biosynthesis of morphinan alkaloids in opium poppy plants remains to be determined.

**Fig 1:**
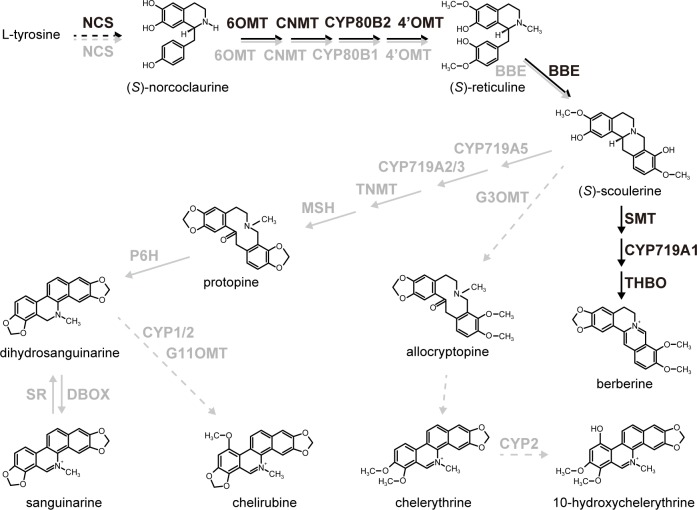
BIA biosynthetic pathways in BIA-producing plants. Black and gray letters show biosynthetic enzymes in *C*. *japonica* and *E*. *californica*, respectively. Broken lines indicate uncharacterized enzyme reactions. NCS, (*S*)-norcoclaurine synthase; 6OMT, (*S*)-norcoclaurine 6-*O*-methyltransferase; CNMT, (*S*)-coclaurine-*N*-methyltransferase; CYP80B1, (*S*)-*N*-methylcoclaurine 3’-hydroxylase; 4’OMT, (*S*)-3’-hydroxy-*N*-methylcoclaurine 4’-*O*-methyltransferase; BBE, berberine bridge enzyme; SMT, (*S*)-scoulerine 9-*O*-methyltransferase of *C*. *japonica*; CYP719A1, (*S*)-canadine synthase; THBO, (*S*)-tetrahydroprotoberberine oxidase; CYP719A5, (*S*)-cheilanthifoline synthase; CYP719A2/3, (*S*)-stylopine synthase; TNMT, (*S*)-tetrahydroprotoberberine cis-*N*-methyltransferase, MSH, (*S*)-*N*-methylstylopine 14-hydroxylase; P6H, protopine 6-hydroxylase; DBOX, dihydrobenzophenanthridine oxidase; SR, sanguinarine reductase; G3OMT, (*S*)-scoulerine 9-*O*-methyltransferase of California poppy; G11OMT, putative 10-hydroxydihydrosanguinarine 10-*O*-methyltransferase. CYP1 and CYP2 are putative dihydrobenzophenanthridine 10-hydroxylases.

In this study, we used the California poppy as a model of BIA biosynthesis in Papaveraceae. This species has common biosynthetic pathways that produce BIAs (either reticuline or some protoberberines) from norcoclaurine, although sanguinarine is the primary alkaloid in California poppy (Fig 1). Importantly, genes encoding biosynthetic enzymes that convert (*S*)-reticuline to sanguinarine have been isolated and characterized [18–24]. Furthermore, generation of stable transformants is easy in this plant species [10].

Thus, we evaluated the *in vivo* function of CjWRKY1 in transgenic California poppy cells by overexpression. Overexpression of the *CjWRKY1* gene induced a clear increase in the expression of several genes encoding BIA biosynthetic enzymes and caused a large accumulation of several BIAs in the medium. Our results suggested that CjWRKY1 functioned in the regulation of BIA biosynthesis in California poppy plants. Correlations between the expression of genes encoding biosynthetic enzymes and alkaloid accumulations in transgenic cultured cells and the diversification of the functional regulation of transcription factors in BIA biosynthesis in BIA-producing plant species are discussed.

## Materials and methods

### Plant materials

Suspension-cultured California poppy cells were grown on a gyratory shaker (90 rpm) at 23°C in the dark in Linsmaier-Skoog (LS) [25] medium (pH 5.7) containing 3% sucrose, 10 μM 1-naphthylacetic acid (NAA), and 1 μM benzyladenine (BA).

### Vector construction

The full-length cDNA of *CjWRKY1* (accession number: AB267401) with *Bam*HI/*Sac*I restriction sites was prepared by PCR and cloned into a pGEM-T Easy vector (Promega, Madison, WI, USA). After the nucleotide sequence was confirmed, the obtained plasmid was digested with *Bam*HI and *Sac*I. The resulting fragment was introduced into a pBIE binary vector [10] that was digested with *Bam*HI and *Sac*I.

### Transformation of California poppy cells

Petioles of *E*. *californica* plants (cultivar, Hitoezaki, Takii Seed Co., Ltd., Kyoto, Japan) were cut into 5–10 mm segments and used for *Agrobacterium tumefaciens* (LBA4404)-mediated transformation as previously described [10]. After approximately 7 months of selection on agar medium containing 150 μg/ml kanamycin and 200 μg/ml cefotaxime, the obtained cells were transferred into liquid LS medium and cultured every 2–3 weeks.

### Quantitative RT-PCR analysis

Total RNA was extracted from suspension-cultured cells with an RNeasy Plant Mini Kit (Qiagen, Hilden, Germany) to determine the expression of *CjWRKY1* and other genes encoding biosynthetic enzymes. Single-stranded cDNAs were synthesized from 1 μg of total RNA with a PrimeScript RT reagent Kit with gDNA Eraser (Takara Bio, Shiga, Japan). Quantitative RT-PCR was performed with specific primer pairs (S1 Table) using iQ^TM^ SYBR Green Supermix and a CFX96 Real-Time PCR Detection System (Bio-Rad, Hercules, CA, USA). The PCR conditions and data calculation method were as described in previous reports [10]. The relative expression levels were values standardized to that of the *β*-actin gene as the internal control, and the expression level of the VC7-1 sample was set at 1. Box plots were created using KaleidaGraph 4.5 software (HULINKS, Tokyo, Japan), with the whiskers representing the highest and lowest values, except for statistical outliers. The box and the band in the box represent the first and third quartiles, and the median, respectively.

### Metabolite analysis

Approximately 1 g fresh weight (FW) of transgenic California poppy cells was inoculated in 20 ml of LS medium and cultured for 5 days for analysis. Approximately 200 mg of fresh cells was ground and extracted in 800 μl of methanol containing 0.05 N HCl at room temperature for 4 h, and the supernatant was prepared for metabolite analysis. Culture medium samples were concentrated to 3 ml with Sep-Pak plus C18 cartridges (Waters, Milford, MA, USA). All samples were analyzed with an LC-MS 2020 (Shimadzu, Kyoto, Japan) under the following conditions: column, TSKgel 80-TM (4.6×250 mm; TOSOH, Tokyo, Japan); acetonitrile/H_2_O solvent gradient containing 1% acetic acid, 30–45% (v/v) for 55 min; UV detection, absorbance measurement at 280 nm with an SPD-20A detector (Shimadzu); flow speed, 0.5 ml/min; column temperature, 40°C; analytical mode, SIM-SCAN (+); and Q array voltage, 100–150 V. The content of each alkaloid was quantified in terms of sanguinarine equivalents using a standard curve derived from an authentic sample. Each UV peak was identified by direct comparison with peaks from standard chemicals. Authentic standards of sanguinarine and chelerythrine were purchased from Sigma-Aldrich, Inc. (St. Louis, MO, USA) and Wako Pure Chemical Industries, Ltd. (Osaka, Japan), respectively. (*R*, *S*)-reticuline was a gift from Mitsui Chemicals, Inc. (Tokyo, Japan). Protopine and allocryptopine were from the collection of Dr. K. Iwasa. Chelirubine and 10-hydroxychelerythrine were identified based on their retention time and mass-to-charge ratio (m/z), as previously described [26]. Box plots are shown as described in the quantitative RT-PCR section.

### Immunoblot analysis

Total protein extraction from cultured California poppy and *C*. *japonica* cells was performed as previously described [27]. Total protein samples (25 μg) were separated by 12% SDS-polyacrylamide gel electrophoresis and then transferred to a polyvinylidene difluoride membrane. The WRKY proteins were detected using anti-CjWRKY1 peptide antibodies [27] (prepared by MBL, 1:1000). After the first round of immunoblotting, the membrane was stripped and re-used for a second immunoblot assay using anti-α-tubulin antibody (T5168; Sigma, St. Louis, MO, USA).

## Results

### Overexpression of *CjWRKY1* in California poppy cells

**Fig 2:**
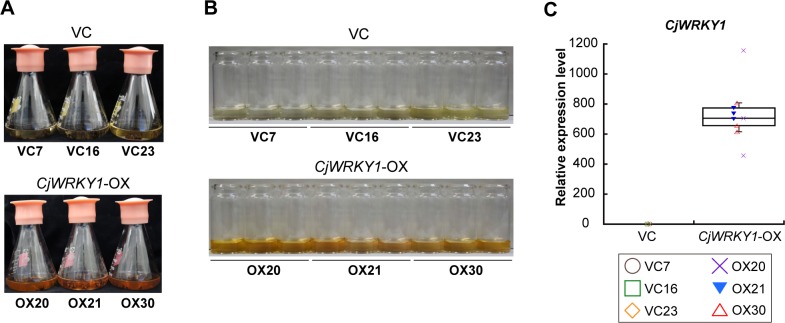
*CjWRKY1*-overexpressing transgenic California poppy cells were established. (A) Three transgenic cell lines were cultured for 5 days. (B) The medium from *CjWRKY1*-OX cell lines showed clear brown pigmentation. (C) Constitutive expression of *CjWRKY1* in transgenic cell lines. The transcript level of *CjWRKY1* was determined by quantitative RT-PCR. Nine biological replicates were used of each cell line.

Effects of overexpression of the *CjWRKY1* gene as a transcriptional activator of BIA biosynthesis were examined in heterologous California poppy cells. Segments of California poppy petioles were transformed with the *CjWRKY1*-overexpression (*CjWRKY1*-OX) vector containing the cauliflower mosaic virus (CaMV) 35S promoter with enhancer elements for constitutive expression with *A*. *tumefaciens* LBA4404 or a control vector (VC) containing the *β-*glucuronidase (*GUS*) gene. After selection with kanamycin, 20–30 transformants were isolated and cultured in liquid culture medium for several months to obtain stable lines. Three cell lines with stable growth were selected for each transformant for further analyses after the genomic PCR analysis of the integration of transgenes. The growth of the selected VC and *CjWRKY1*-OX transgenic cell lines was similar, but the *CjWRKY1*-OX cell lines, compared with the VC cell lines, showed clear pigmentation in the medium (Fig 2A and 2B).

### Transcriptional activation of several genes encoding BIA biosynthetic enzymes by ectopic expression of CjWRKY1

**Fig 3:**
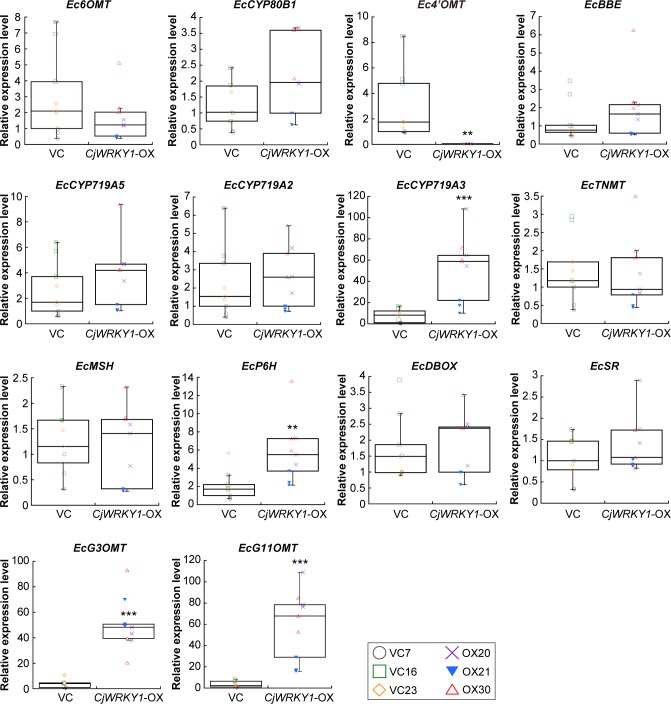
Effects of *CjWRKY1*-overexpression on genes encoding BIA biosynthetic enzymes in transgenic California poppy cell lines. The transcript levels of *Ec6OMT*, *EcCYP80B1*, *Ec4’OMT*, *EcBBE*, *EcCYP719A5*, *EcCYP719A2*, *EcCYP719A3*, *EcTNMT*, *EcMSH*, *EcP6H*, *EcDBOX*, *EcSR*, *EcG3OMT*, and *EcG11OMT* were determined by quantitative RT-PCR. Nine biological replicates were used of each cell line. Asterisks indicate a significant difference compared with VC cell lines (*P < 0.05, **P < 0.01, ***P < 0.001; two-tailed paired Student’s *t*-test).

Quantitative RT-PCR analysis confirmed the high expression of *CjWRKY1* in all *CjWRKY1*-OX cell lines (OX20, OX21, and OX30), compared with VC cell lines (VC7, VC16, and VC23) (Fig 2C). Because of the dispersion of results derived from the variation in cell cultures, we showed the results as a box plot. Further analysis showed few changes in the expression of genes encoding biosynthetic enzymes involved in BIA biosynthesis, i.e., *(S)-norcoclaurine 6-O-methyltransferase* (*Ec6OMT*), *(S)-N-methylcoclaurine 3’-hydroxylase* (*EcCYP80B1*), *berberine bridge enzyme* (*EcBBE*), *(S)-cheilanthiforine synthase* (*EcCYP719A5*), *(S)-stylopine synthase* (*EcCYP719A2*), *(S)-tetrahydroprotoberberine cis-N-methyltransferase* (*EcTNMT*), *(S)-N-methylstylopine 14-hydroxylase* (*EcMSH*), *dihydrobenzophenanthridine oxidase* (*EcDBOX*), and *sanguinarine reductase* (*EcSR*) (Fig 3). However, *(S)-stylopine synthase* (*EcCYP719A3*) and *protopine-6-hydroxylase* (*EcP6H*) increased significantly in *CjWRKY1*-OX cells (Fig 3). Unexpectedly, the *(S)-3'-hydroxy-N-methylcoclaurine 4'-O-methyltransferase* (*Ec4’OMT*) gene showed a dramatic decrease in expression in *CjWRKY1*-OX cells (Fig 3). When four recently isolated novel biosynthetic enzymes (Fig 1), *(S)-scoulerine 9-O-methyltransferase* (*G3OMT*), putative *10-hydroxydihydrosanguinarine 10-O-methyltransferase* (*G11OMT*), and two cytochrome P450s (CYP1 and CYP2), in the biosynthesis of chelirubine, chelerythrine, and 10-hydroxychelerythrine (10-HC) in California poppy [28] (Hori et al., in preparation) were also analyzed in transgenic California poppy cells, we found significant increases of *EcG3OMT* and *EcG11OMT* transcripts and a moderate increase of those of *EcCYP2* in *CjWRKY1*-OX cells, in contrast to decrease in *EcCYP1* transcripts (Fig 3, S1A Fig). This preferential modulation of expression of genes encoding BIA biosynthetic enzymes in California poppy cells was considerably different from that in *C*. *japonica* cells in which CjWRKY1 broadly modulated the expression of genes involved in BIA biosynthesis [11].

**Fig 4:**
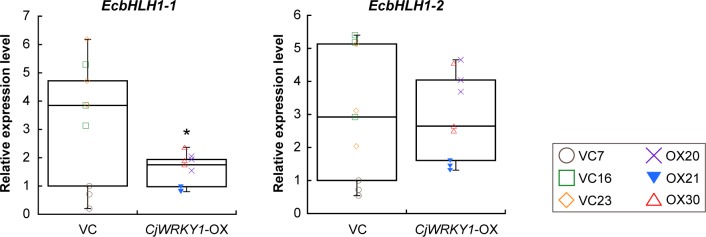
Effects of overexpression of the *CjWRKY1* gene on bHLH transcription factor genes in transgenic California poppy cell lines. The expression levels of *EcbHLH1-1* and *EcbHLH1-2* were determined by quantitative RT-PCR. Nine biological replicates were used of each cell line. An asterisk indicates a significant difference compared with VC cell lines (*P < 0.05; two-tailed paired Student’s *t*-test).

Similar preferential modulation of expression of genes involved in BIA biosynthesis was also observed in California poppy cells when RNA silencing was performed with the bHLH transcription factors EcbHLH1-1 and EcbHLH1-2 [10]. Previous results suggest that EcbHLH1-2 primarily regulates BIA biosynthesis in cultured California poppy cells. However, the effect of *EcbHLH1-1* expression on that of *EcCYP719A3* and *EcTNMT* was much stronger than that of *EcbHLH1-2*, suggesting that EcbHLH1-1 preferentially controls the expression of *EcCYP719A3* and *EcTNMT* genes [10]. When we measured the expression levels of *EcbHLH1-1* and *EcbHLH1-2* in *CjWRKY1*-OX cell lines (Fig 4), the expression of *EcbHLH1-1* significantly decreased in *CjWRKY1*-OX cells, whereas the expression of *EcbHLH1-2* did not.

### Modulation of BIA biosynthesis by overexpression of *CjWRKY1* in California poppy cells

**Fig 5:**
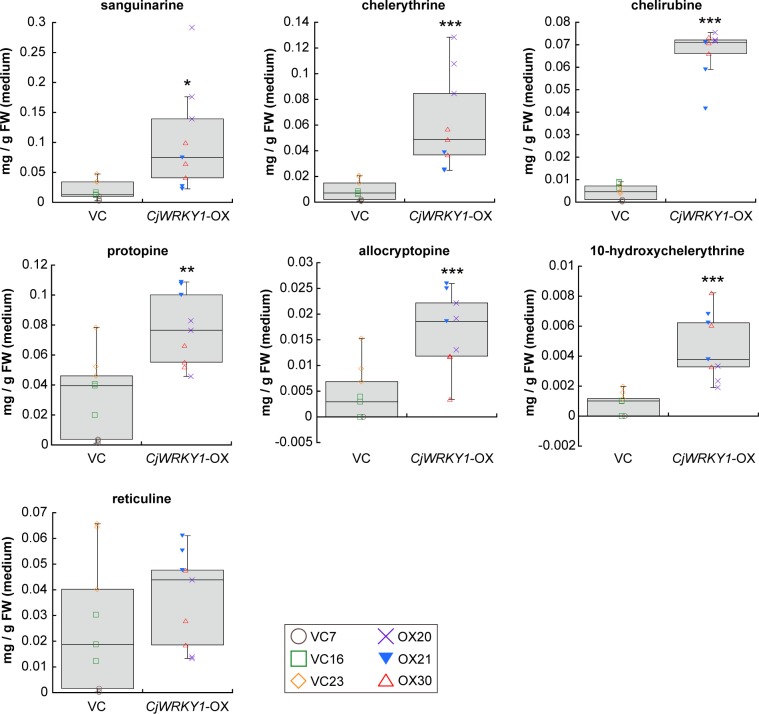
The accumulation of BIAs in the medium from transgenic California poppy cell lines. The contents of sanguinarine, chelerythrine, chelirubine, protopine, allocryptopine, 10-hydroxychelerythrine, and reticuline were estimated based on the peak areas using the standard curve created from an authentic sanguinarine sample. For each cell line, nine biological replicates were used. Asterisks indicate significant differences from VC cell lines (*P < 0.05, **P < 0.01, ***P < 0.001; two-tailed paired Student’s *t*-test).

**Fig 6:**
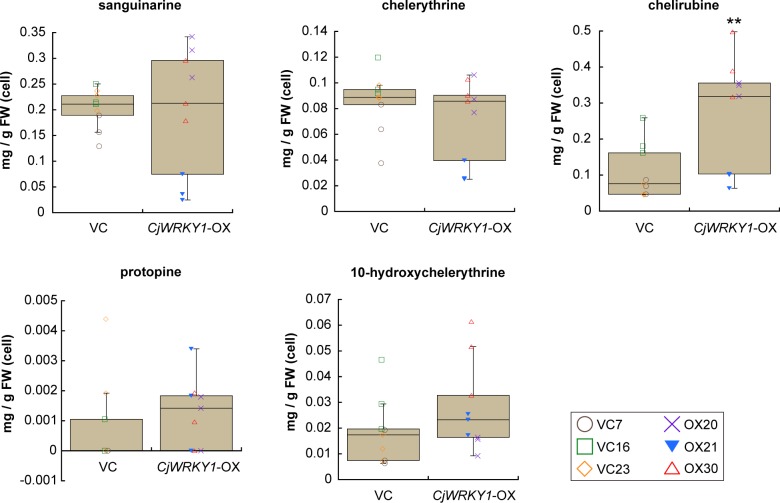
The accumulation of representative BIAs in transgenic cells of California poppy. The contents of sanguinarine, chelerythrine, chelirubine, protopine, and 10-hydroxychelerythrine in cell extract were calculated using the standard curve of sanguinarine. Nine biological replicates were used of cell line. Asterisks indicate significant differences from VC cells (**P < 0.01, two-tailed paired Student’s *t*-test).

To evaluate the effect of modified expression of genes encoding biosynthetic enzymes in BIA biosynthesis, the quantities of alkaloids in culture medium and in cultured cells were determined in terms of sanguinarine equivalents, based on the intensities of their signals. Whereas the alkaloid composition was nearly identical in both cell lines, the amounts of several alkaloids were different, particularly in the culture medium. The accumulation of sanguinarine, chelerythrine, chelirubine, protopine, allocryptopine, and 10-HC markedly increased in the culture medium of *CjWRKY1*-OX cells compared with that in VC cell lines (*P*-values calculated by two-tailed paired Student’s *t*-tests; 0.011 for sanguinarine; 8.1 × 10^−4^ for chelerythrine; 1.5 × 10^−11^ for chelirubine; 0.0016 for protopine; 8.0 × 10^−4^ for allocryptopine; and 1.3 × 10^−4^ for 10-HC), whereas reticuline showed no significant change (*P* = 0.33) (Fig 5). However, between *CjWRKY1*-OX and VC cells, the differences in the amounts of sanguinarine (*P* = 0.86), chelerythrine (*P* = 0.30), protopine (*P* = 0.49), and 10-HC (*P* = 0.20) were much less. Only chelirubine significantly increased in *CjWRKY1*-OX cells compared with VC cells (*P* = 0.0084) (Fig 6). Allocryptopine and reticuline did not accumulate in cultured cells because these two compounds were likely exported to the medium.

When the overall accumulation of alkaloids in the flasks was calculated (S2 Fig), chelirubine and protopine showed marked increases with *CjWRKY1*-OX cell lines (0.10–0.57 mg/g FW of chelirubine, *P* = 0.0012 and 0.046–0.11 mg/g FW of protopine, *P* = 0.0018) compared with VC cell lines (0.048–0.27 mg/g FW of chelirubine and 0.0012–0.080 mg/g FW of protopine). However, no significant differences were detected for sanguinarine (*P* = 0.28), chelerythrine (*P* = 0.11), and 10-HC (*P* = 0.097).

**Fig 7:**
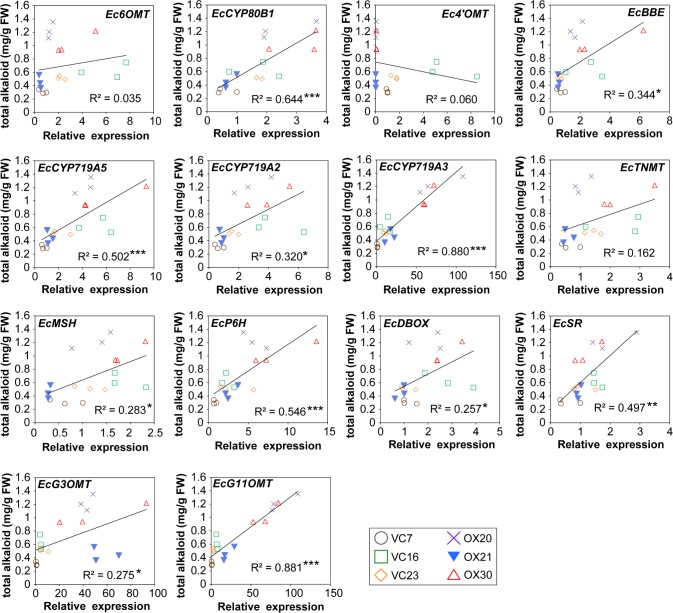
Correlation analysis of the expression of genes encoding biosynthetic enzymes and the accumulation of total alkaloids in flasks growing transgenic California poppy cells. Asterisks indicate significant correlations (df = 18; *P < 0.05, **P < 0.01, ***P < 0.001).

Finally, we calculated the correlation between the expression of genes encoding biosynthetic enzymes and the overall accumulation of alkaloids (sanguinarine, chelerythrine, chelirubine, protopine, 10-HC, and allocryptopine) in the flasks (Fig 7, S1B Fig). The correlation between expression and the accumulation of each alkaloid was also calculated (S3–S8 Figs). *EcCYP719A3* and *EcG11OMT* expression showed the highest correlation with alkaloid accumulation, which suggested that EcCYP719A3 and EcG11OMT might be involved in the rate-limiting step in BIA biosynthesis in California poppy. However, *Ec4’OMT* showed little correlation with alkaloid accumulation, suggesting less contribution to the biosynthesis.

Considering the variation of each cell line, we further analyzed the data of gene expression and metabolite accumulation using one-way ANOVA with post-hoc Tukey-Kramer test (S9 and S10 Figs). This analysis revealed that the expression levels of *EcCYP719A3*, *EcG3OMT* and *EcG11OMT* in *CjWRKY1*-OX cells were higher than those in VC cells. Metabolite analyses also revealed the same results, as shown in Figs 5 and 6 and S2 Fig, although the difference in accumulation of sanguinarine in culture medium was only slight between VC and *CjWRKY1*-OX cell lines (likely caused by the variation of cell cultures). These results from two different statistical analyses indicated that the overexpression of *CjWRKY1* facilitated the production of BIAs in California poppy cells.

## Discussion

The regulation of gene expression of biosynthetic enzymes by transcription factors is a very powerful strategy to increase metabolite production. However, each biosynthetic pathway has specific transcription factors (e.g., bHLH, MYB, and WD40 in phenylpropanoid biosynthesis and WRKY, ERF, and MYC in the biosynthesis of some alkaloids), and each plant species has additional specific transcription factors that regulate these pathways [29, 30]. In this work, we examined the ectopic expression of *CjWRKY1*, a BIA regulator in Ranunculaceae, in the biosynthesis of BIAs in California poppy, a Papaveraceae. Overexpression of *CjWRKY1* resulted in increased accumulation of metabolites in the culture medium. Further analysis of gene expression indicated preferential activation/modulation of gene expression in BIA biosynthesis. This preferential modulation of gene expression was due to the heterologous expression of this transcription factor, suggesting that although the biosynthetic pathways in each plant species are similar, each gene, including a promoter region, might be different among plant species. Because our preliminary analysis of biosynthetic enzyme gene promoters using the draft genome sequence of California poppy did not indicate a correlation between the number of *cis*-elements and gene expression, more accurate characterization of promoter sequences and of the target sequences of CjWRKY1 is necessary in the future. Furthermore, a recent study showed that microRNAs might be involved in the regulation of BIA biosynthesis in opium poppy [31]. The interactions between WRKY and bHLH transcription factors and microRNAs in a transcriptional network of BIA biosynthesis should also be investigated in future study.

Although CjWRKY1 was identified as a comprehensive regulator of almost all genes encoding berberine biosynthetic enzymes in *C*. *japonica* [11], the ectopic expression of *CjWRKY1* in *E*. *californica* resulted in the increase in the expression of only *EcCYP719A3*, *EcP6H* and two novel *OMT* (*EcG3OMT* and *EcG11OMT*) genes (Fig 3). These results suggested the functional diversification of WRKY transcription factors in BIA biosynthesis. In fact, a similar diversification in the regulatory functions of CjbHLH1 and EcbHLH1-1/EcbHLH1-2 is observed [9, 10]. Furthermore, Apuya and colleagues report that the heterologous expression of *AtWRKY1* from *A*. *thaliana* in California poppy cells results in increased accumulation of several BIAs [13]. Intriguingly, PsWRKY from *P*. *somniferum*, a potent transcriptional activator of BIA biosynthesis in opium poppy, belongs to the same group as AtWRKY1, because both WRKY proteins have two WRKY domains [12]. CjWRKY1 and PsWRKY homologs from *E*. *californica* must be isolated to investigate their *in vivo* function and the functional relationship between them in BIA biosynthesis.

Whereas Apuya and colleagues report that the overexpression of *AtWRKY1* in California poppy cells increases the accumulation of BIAs up to approximately 30-fold [13], the overexpression of *CjWRKY1* increased the accumulation of chelirubine and protopine in both medium and cells only up to 3- to 4-fold (S2 Fig). Furthermore, the accumulation of several BIAs, except for chelirubine, in cells showed no difference between VC and *CjWRKY1*-OX cell lines (Fig 6). One possible explanation is that the post-translational regulatory mechanism(s) of these transcription factors might have a large effect on alkaloid productivity. Our previous report suggests that the stability of the CjWRKY1 protein is regulated by post-translational modifications such as protein phosphorylation and degradation in *C*. *japonica* [27]. In the immunoblot analysis with anti-CjWRKY1 antibodies, accumulation of overexpressed CjWRKY1 protein was undetectable and predicted CjWRKY1 homolog protein(s) might not have been altered in California poppy (S11 Fig), although the transcript level of the ectopic *CjWRKY1* gene might be sufficient for detection, according to the result of quantitative RT-PCR (Fig 2C). The greater effect of AtWRKY1 on the productivity of BIAs in California poppy cells may indicate that the post-translational modification(s) of AtWRKY1 protein is not essential. To facilitate metabolic engineering in future studies, the determination of more detailed protein modification mechanisms leading to increased stability of the CjWRKY1 protein will be useful.

The correlation analyses indicated that the expression of *EcCYP719A3*, *EcP6H*, and *EcG11OMT*, which increased significantly in *CjWRKY1*-OX cell lines, and also that of *EcCYP80B1*, *EcBBE*, *EcCYP719A5*, *EcCYP719A2*, *EcMSH*, *EcDBOX*, *EcSR*, and *EcCYP2*, which did not increase significantly between VC and *CjWRKY1*-OX cell lines, was significantly correlated with alkaloid accumulation in flasks containing transgenic California poppy cells (Fig 7, S1B Fig). The highest correlations between *EcCYP719A3* expression and the accumulations of sanguinarine and chelerythrine suggested that EcCYP719A3 functioned in a key step of their biosynthetic pathways (S3 and S4 Figs). The highest correlation between the expression of *EcG11OMT* and the accumulation of chelirubine also coincided with the activity of EcG11OMT, which catalyzes the *O*-methylation of 10-hydroxybenzophenanthridine alkaloids in chelirubine biosynthesis (S5 Fig) [28]. Correlation analysis also revealed significant correlations between *EcG3OMT* expression and the accumulation of protopine and *EcCYP2* expression and the accumulation of 10-HC, suggesting the involvement of EcG3OMT and EcCYP2 in the biosynthesis of protopine and 10-HC, respectively (S6 and S7 Figs). Our functional characterization reveals that EcCYP2 is involved in the 10-hydroxylation of dihydrobenzophenanthridine alkaloids (Hori et al., in preparation), whereas the relationship between EcG3OMT and protopine biosynthesis remains unclear. By contrast, the expression of *Ec4’OMT* clearly decreased in *CjWRKY1*-OX cell lines and showed no correlation with alkaloid accumulation, which indicated that the steps involving Ec4’OMT might not be rate-limiting in these cell lines (Figs 4 and 7). Recently, elicitor-induced overproduction of secondary metabolites was found to be involved in negative feedback regulation that prevented their overproduction through the inhibition of elicitor-responsive phospholipase A2 (PLA2) activities [32]. Hence, the high concentration of alkaloids in the culture medium might have affected the expression of biosynthetic enzymes and the metabolite productivity of *CjWRKY1*-OX transgenic cultured cells. To examine the effect of metabolites on gene expression, we treated VC cells (VC23) with 25 μM sanguinarine for 24 h and measured the gene expression of the biosynthetic enzymes (S12 Fig). As a result, the expression of genes encoding biosynthetic enzymes decreased significantly, albeit moderately. In a previous report, the greatest inhibitory effect of chelirubine and 10-HC was on the activity of PLAs [32]; thus, the decrease in the expression of genes encoding biosynthetic enzymes, particularly *Ec4’OMT*, might be induced by multiple benzophenanthridine alkaloids. Although the expression of *Ec4’OMT* decreased markedly, alkaloid productivity was not affected, suggesting that other OMT(s), such as Ec6OMT, complement the 4’OMT activity [33].

Our findings indicate that at least two transcription factors, WRKY1 and bHLH1, are substantially involved in the regulation of BIA biosynthesis, whereas the analysis of *EcbHLH1* expression in *CjWRKY1*-OX cells did not reveal a functional relationship between WRKY and bHLH transcription factors (Fig 4). Functional interactions between transcriptional regulators including not only WRKY1 and bHLH1 but also novel transcription factor(s) and interacting factor(s) is thought to be required for the appropriate control of alkaloid productivity. Recent advances in whole-genome sequencing technologies should aid in determination of how transcriptional networks that spatiotemporally regulate BIA biosynthesis formed and evolved in specific plants.

## Supporting information

S1 Fig Expression analysis of genes encoding novel biosynthetic enzymes in transgenic California poppy cells. (A) The transcript levels of *EcCYP1* and *EcCYP2* were determined by quantitative RT-PCR. Nine biological replicates were used of each cell line. (B) Correlation analysis between the expression of *EcCYP1* and *EcCYP2* and the accumulation of total alkaloids in flasks containing transgenic California poppy cells. Asterisks indicate significant differences from VC cell lines (*P < 0.05; Student’ s *t*-test).
(PDF)

S2 Fig The total accumulation of BIAs in both cells and the medium of transgenic California poppy cells. The contents of sanguinarine, chelerythrine, chelirubine, protopine, and 10-hydroxychelerythrine were estimated using the standard curve of authentic sanguinarine. Nine biological replicates were used of each cell line. Asterisks indicate significant differences from VC cell lines (**P < 0.01; Student’s *t*-test).
(PDF)

S3 Fig Correlation analysis between the expression of genes encoding biosynthetic enzymes and the accumulation of sanguinarine in flasks with transgenic California poppy cells. Asterisks indicate a significant correlation (df = 18; *P < 0.05, **P < 0.01, ***P < 0.001).
(PDF)

S4 Fig Correlation analysis between the expression of genes encoding biosynthetic enzymes and the accumulation of chelerythrine in flasks containing transgenic California poppy cells. Asterisks indicate a significant correlation (df = 18; *P < 0.05, **P < 0.01, ***P < 0.001).
(PDF)

S5 Fig Correlation analysis between the expression of genes encoding biosynthetic enzymes and the accumulation of chelirubine in flasks with transgenic California poppy cells. Asterisks indicate a significant correlation (df = 18; *P < 0.05, **P < 0.01, ***P < 0.001).
(PDF)

S6 Fig Correlation analysis between the expression of genes encoding biosynthetic enzymes and the accumulation of protopine in flasks with transgenic California poppy cells. Asterisks indicate a significant correlation (df = 18; **P < 0.01).
(PDF)

S7 Fig Correlation analysis between the expression of genes encoding biosynthetic enzymes and the accumulation of 10-HC in flasks with transgenic California poppy cells. Asterisks indicate a significant correlation (df = 18; *P < 0.05).
(PDF)

S8 Fig Correlation analysis between the expression of genes encoding biosynthetic enzymes and the accumulation of allocryptopine in flasks with transgenic California poppy cells. Asterisks indicate a significant correlation (df = 18; *P < 0.05, **P < 0.01).
(PDF)

S9 Fig One-way ANOVA of expression of biosynthetic enzyme-encoding genes in transgenic California poppy cells. The transcript levels of genes were determined by quantitative RT-PCR. The value is the average of results from three biological replicates. The data are shown as the mean ±s.d.; significance was determined with one-way ANOVA with post-hoc Tukey-Kramer test.
(PDF)

S10 Fig One-way ANOVA of metabolites in transgenic California poppy cell lines. The content of representative alkaloids in the medium (A), cells (B), and both cells and the medium (C) was calculated using the standard curve of sanguinarine. The value is the average of results from three biological replicates. The data are shown as the mean ±s.d.; significance was determined with one-way ANOVA with post-hoc Tukey-Kramer test.
(PDF)

S11 Fig Accumulation of CjWRKY1 protein was not observed in transgenic California poppy cells. Total protein extracts from VC (A) and *CjWRKY1*-OX (B) cultured cells were used for immunoblot analysis with anti-CjWRKY1 peptide antibodies and anti-α-tubulin antibody. C: the extract of *C*. *japonica* cultured cells served as a positive control. An asterisk and arrow indicate nonspecific protein (possible CjWRKY1 homolog) and CjWRKY1 protein, respectively.
(PDF)

S12 Fig Treatment of VC cells with 25 μM sanguinarine affected the expression levels of genes encoding BIA biosynthetic enzymes. The transcript levels of *Ec6OMT*, *Ec4’OMT*, *EcCYP719A5*, *EcTNMT*, and *EcP6H* were determined by quantitative RT-PCR. The relative expression levels were estimated by the standard curve method with three technical replicates and were standardized to the expression of the *β-actin* gene as the internal control. The average value of the control (0.25% methanol) was set as 1. The data are shown as the mean ±s.d.; *P < 0.05. **P < 0.01, Student’s *t*-test.
(PDF)

S1 Table Primers for quantitative RT-PCR of target genes. (PDF)
