# Supplementary figures and images for: Modulation of benzylisoquinoline alkaloid biosynthesis by heterologous expression of CjWRKY1 in Eschscholzia californica cells
Source: PLoS One. 2017 Oct 27;12(10):e0186953. doi: 10.1371/journal.pone.0186953 (PMC5659775; doi:10.1371/journal.pone.0186953)

**A**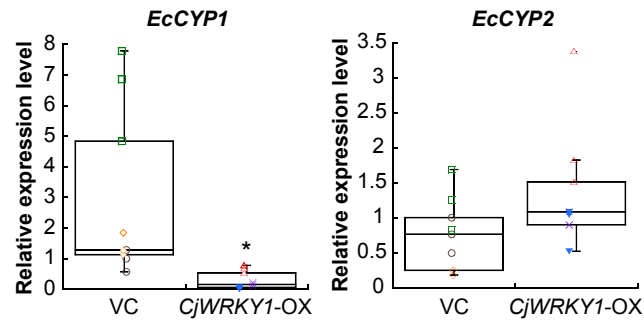**B**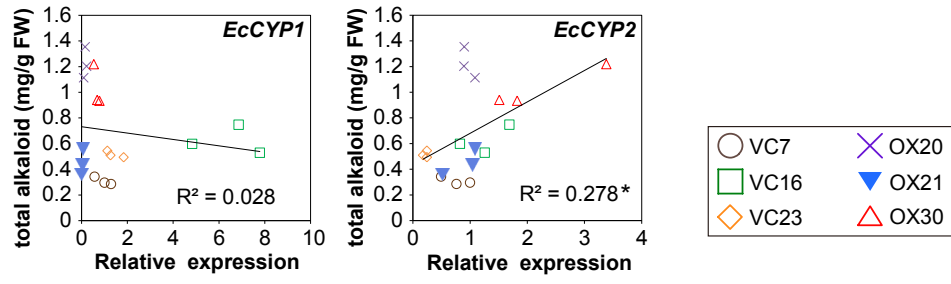

Supplement: S1 Fig — (A) The transcript levels of EcCYP1 and EcCYP2 were determined by quantitative RT-PCR. Nine biological replicates were used of each cell line. (B) Correlation analysis between the expression of EcCYP1 and EcCYP2 and the accumulation of total alkaloids in flasks containing transgenic California poppy cells. Asterisks indicate significant differences from VC cell lines (*P < 0.05; Student’ s t-test). (PDF) [file pone.0186953.s001.pdf]

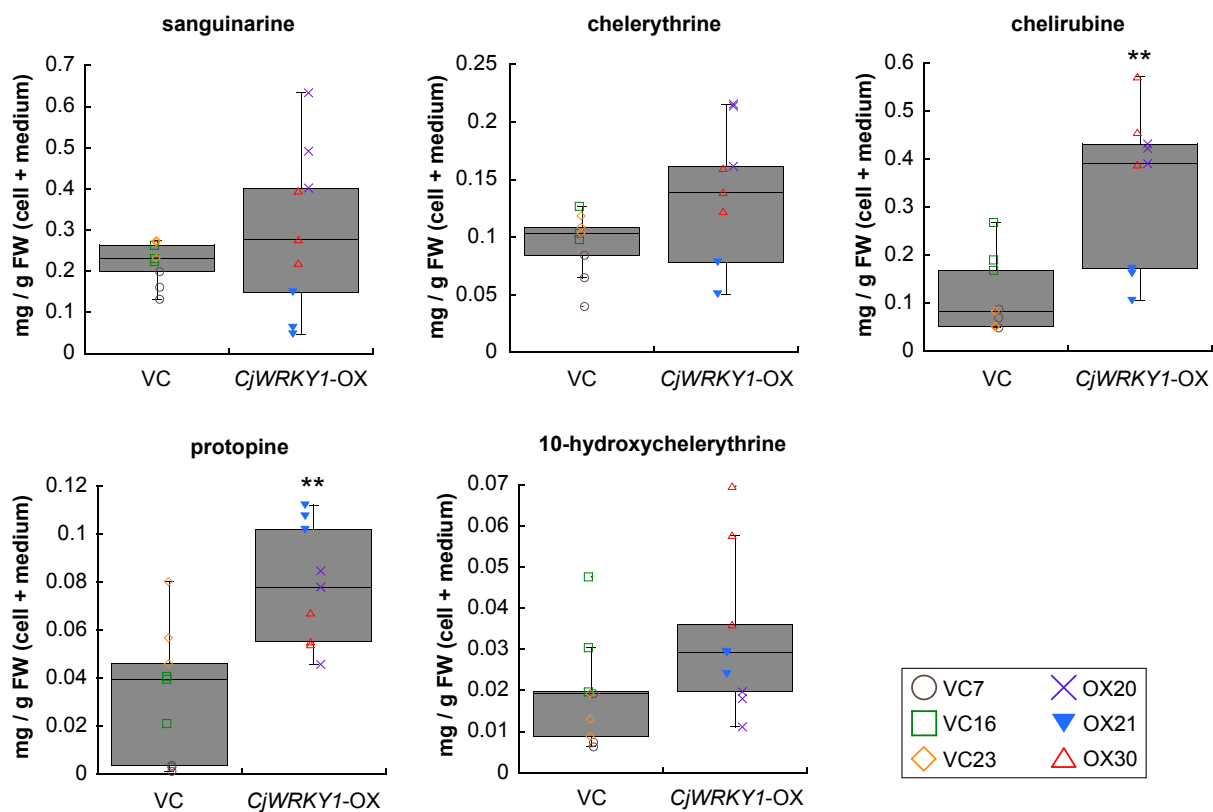

Supplement: S2 Fig — The contents of sanguinarine, chelerythrine, chelirubine, protopine, and 10-hydroxychelerythrine were estimated using the standard curve of authentic sanguinarine. Nine biological replicates were used of each cell line. Asterisks indicate significant differences from VC cell lines (**P < 0.01; Student’s t-test). (PDF) [file pone.0186953.s002.pdf]

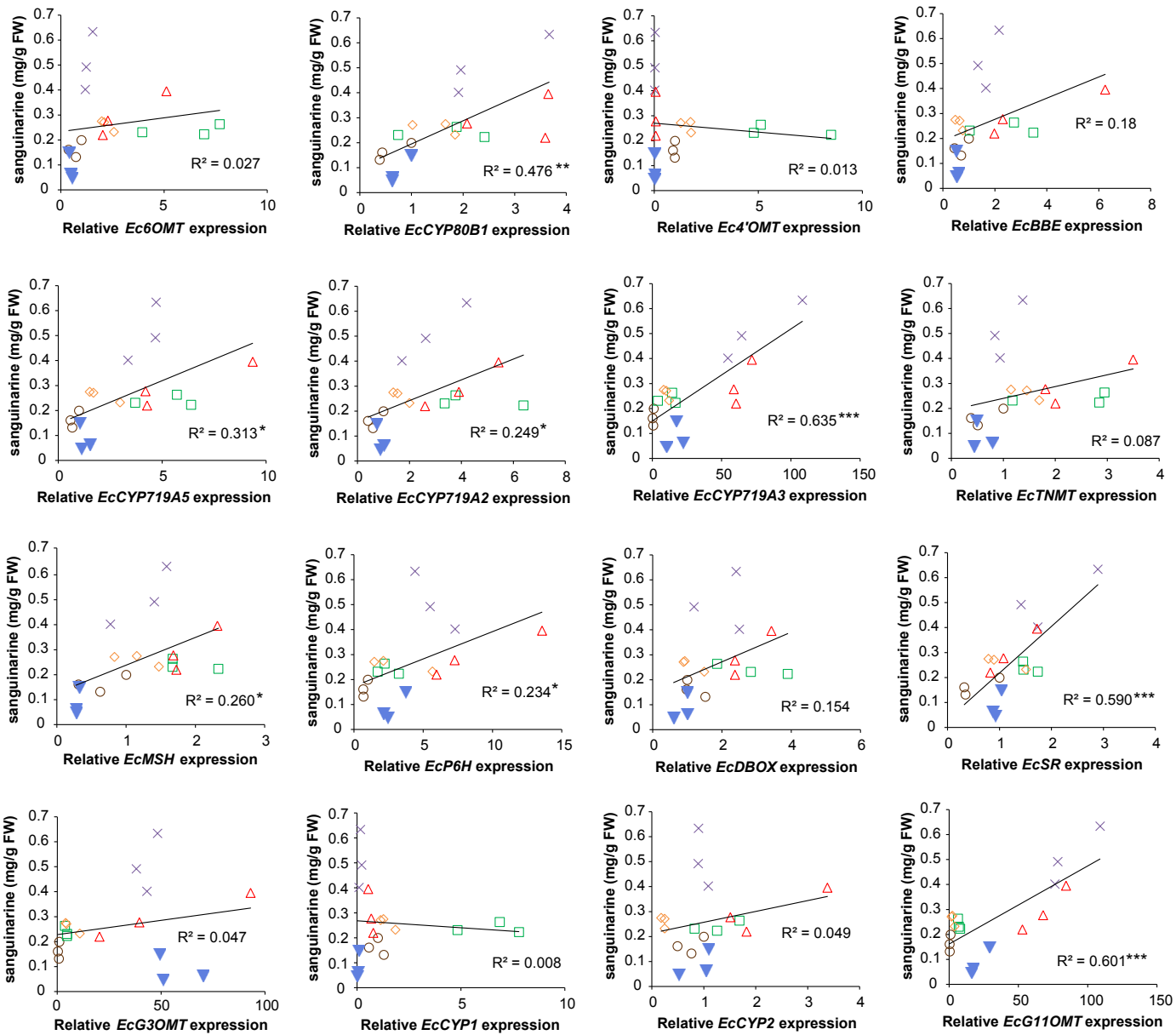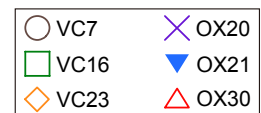

Supplement: S3 Fig — Asterisks indicate a significant correlation (df = 18; *P < 0.05, **P < 0.01, ***P < 0.001). (PDF) [file pone.0186953.s003.pdf]

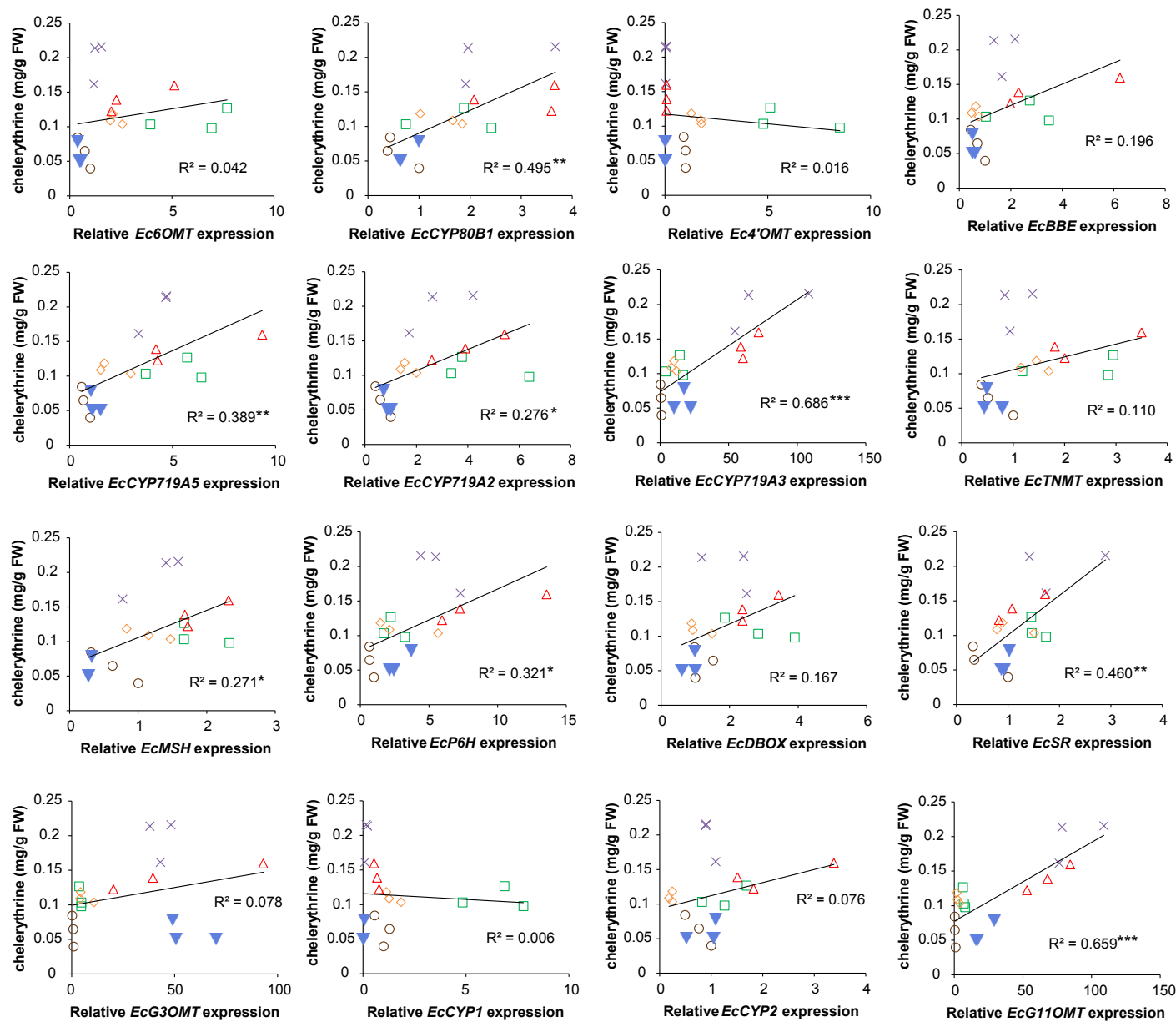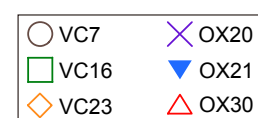

Supplement: S4 Fig — Asterisks indicate a significant correlation (df = 18; *P < 0.05, **P < 0.01, ***P < 0.001). (PDF) [file pone.0186953.s004.pdf]

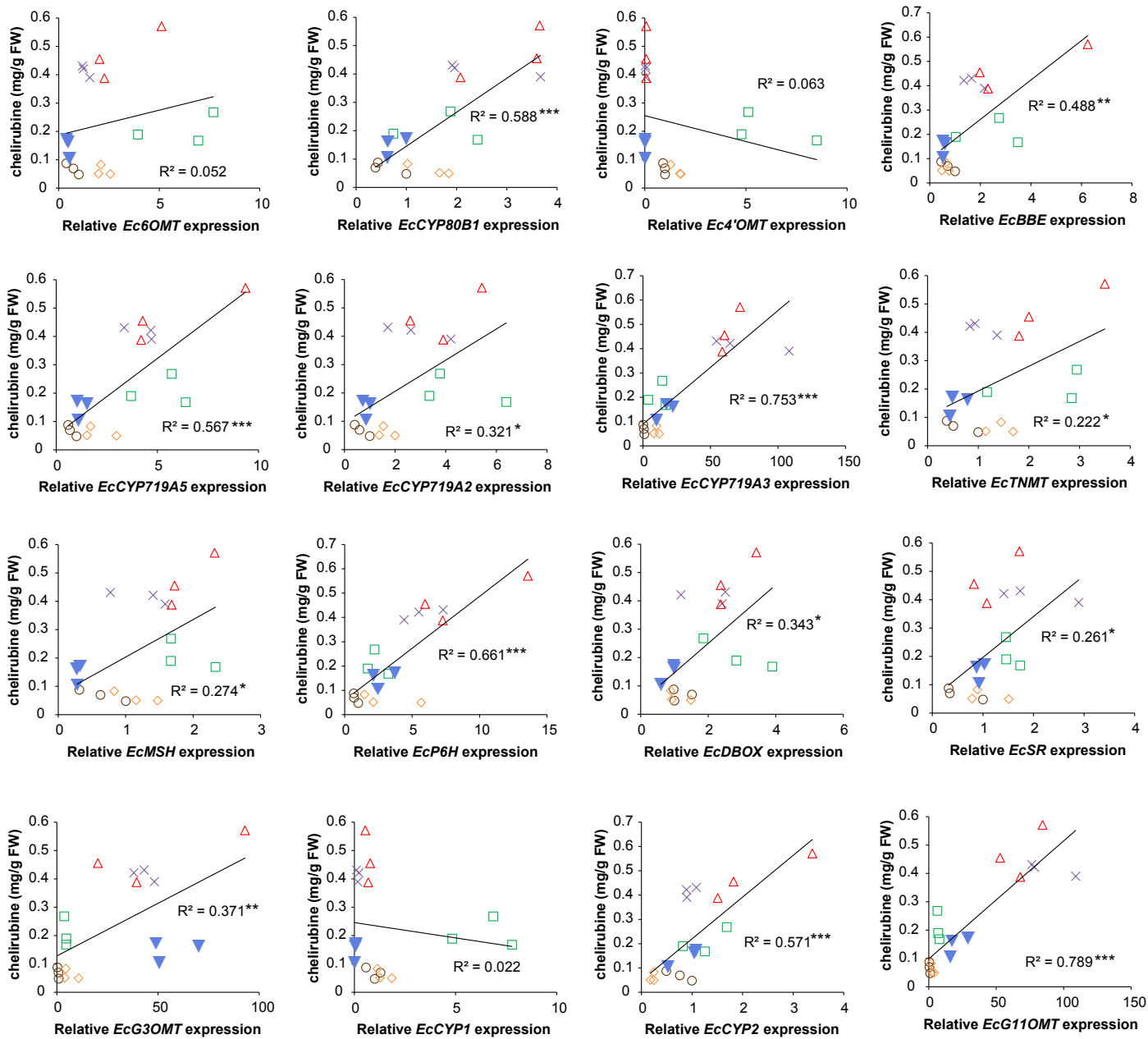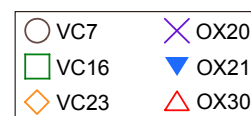

Supplement: S5 Fig — Asterisks indicate a significant correlation (df = 18; *P < 0.05, **P < 0.01, ***P < 0.001). (PDF) [file pone.0186953.s005.pdf]

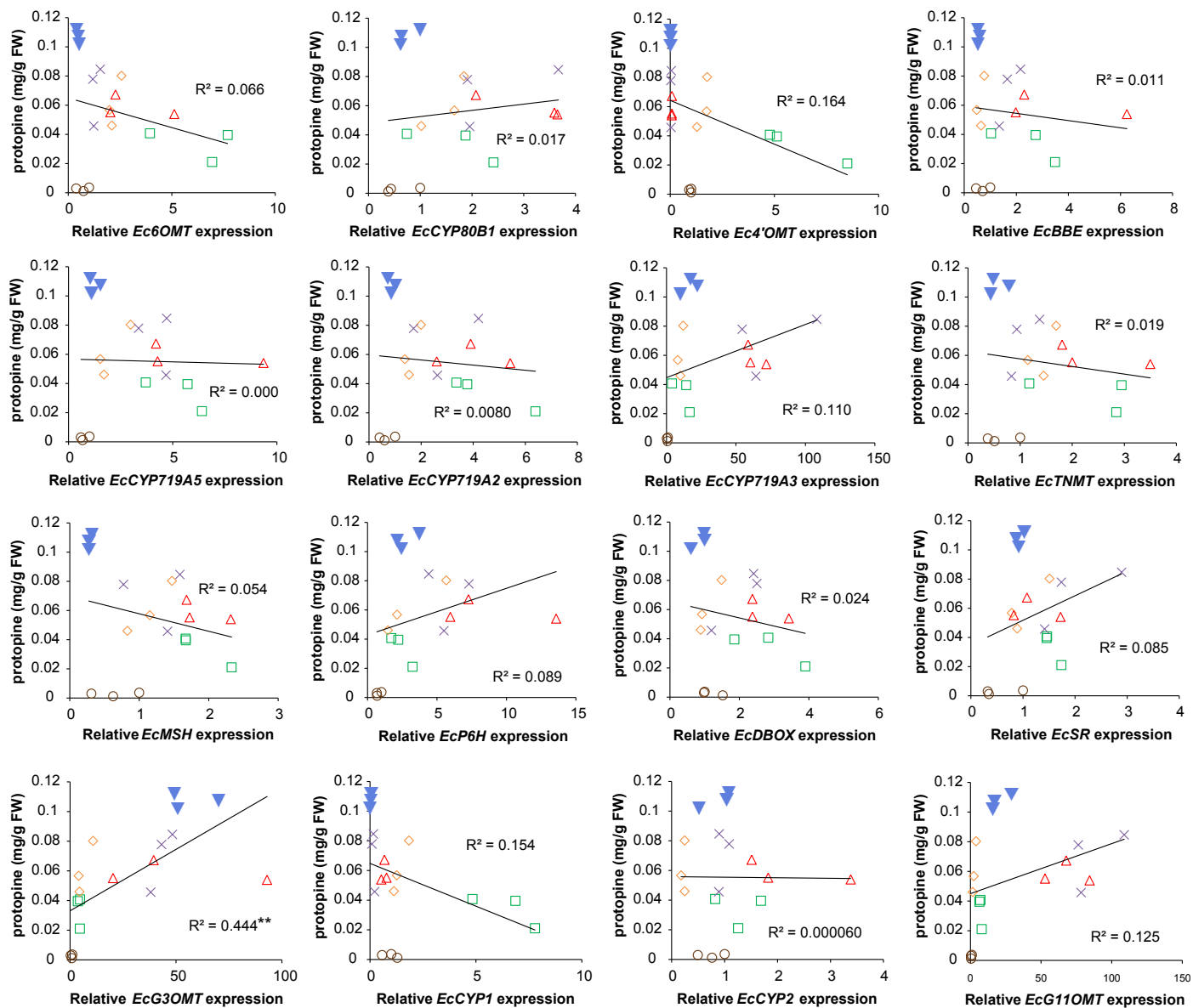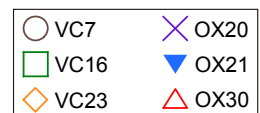

Supplement: S6 Fig — Asterisks indicate a significant correlation (df = 18; **P < 0.01). (PDF) [file pone.0186953.s006.pdf]

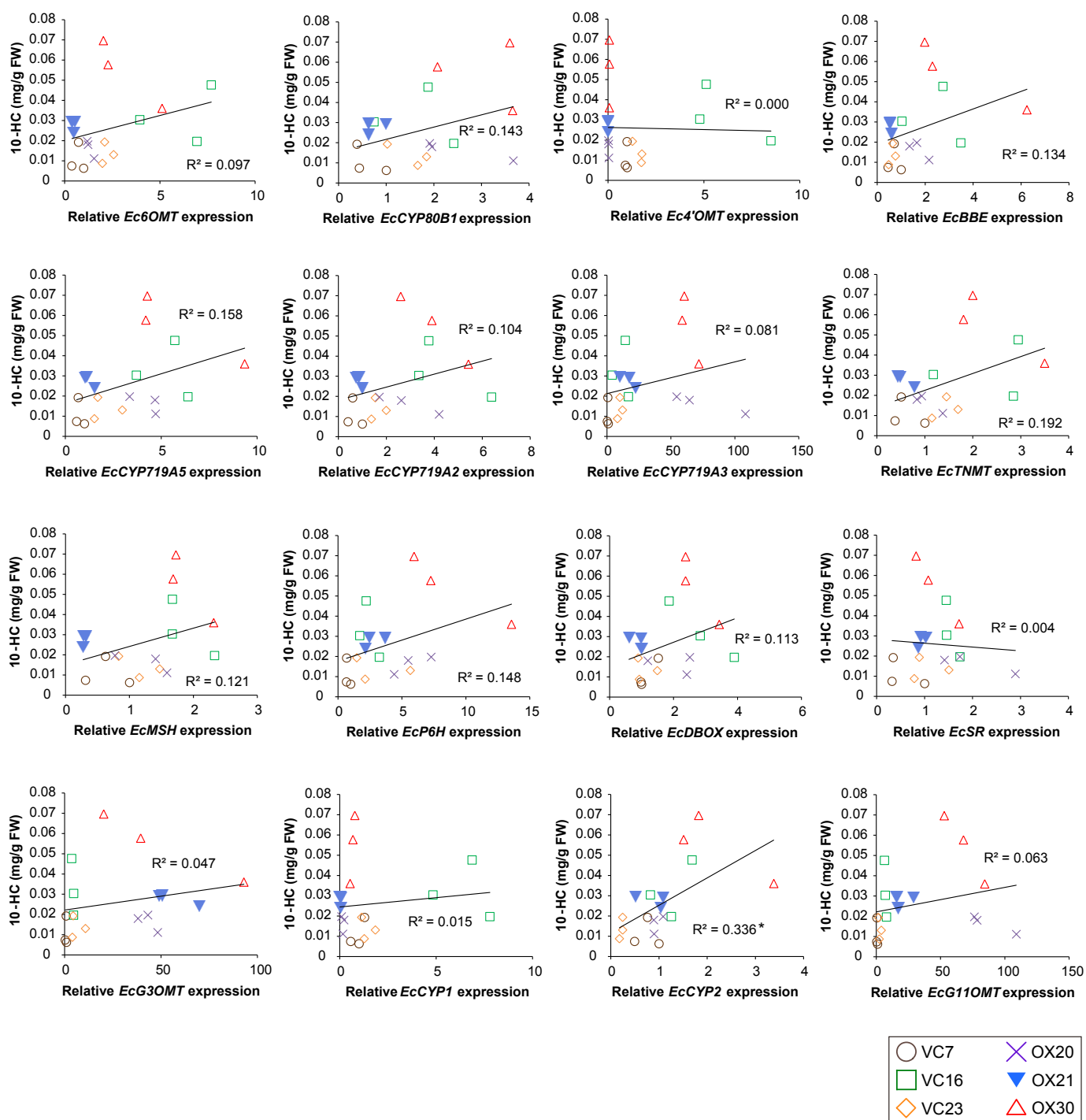

Supplement: S7 Fig — Asterisks indicate a significant correlation (df = 18; *P < 0.05). (PDF) [file pone.0186953.s007.pdf]

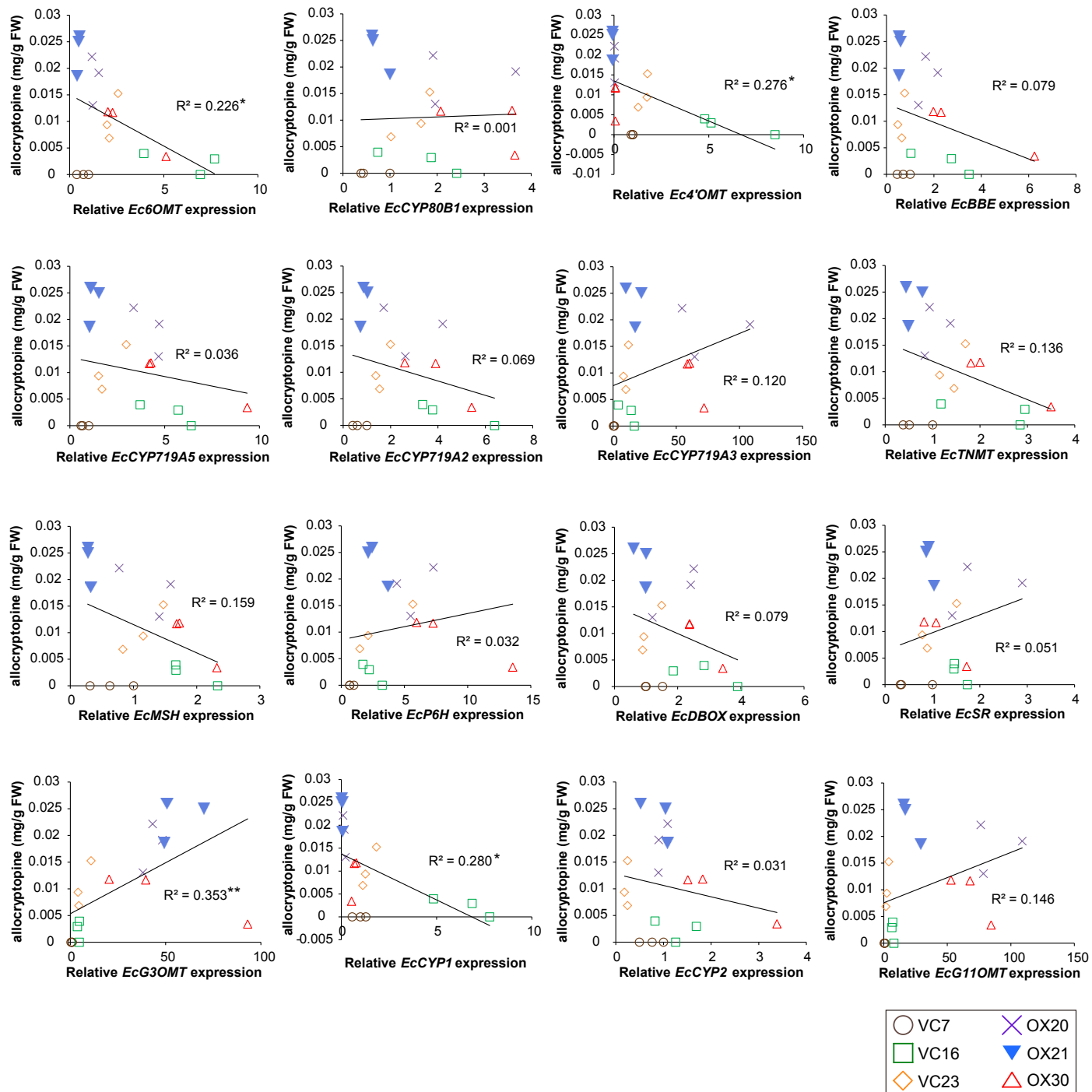

Supplement: S8 Fig — Asterisks indicate a significant correlation (df = 18; *P < 0.05, **P < 0.01). (PDF) [file pone.0186953.s008.pdf]

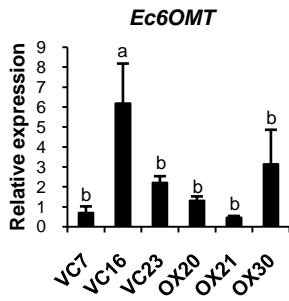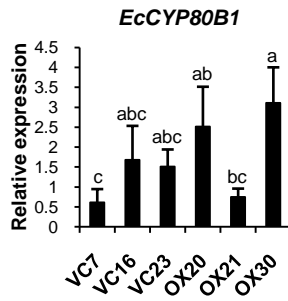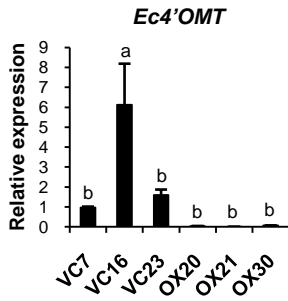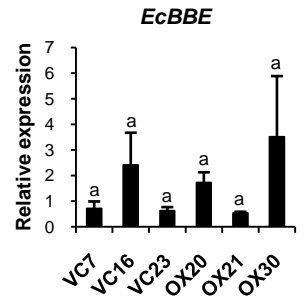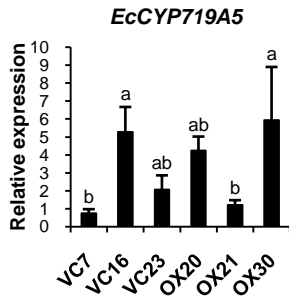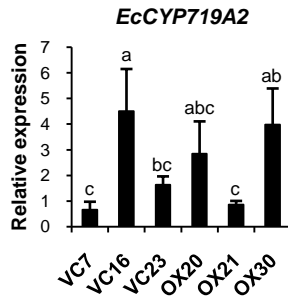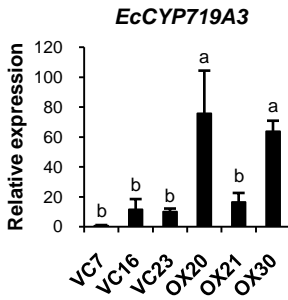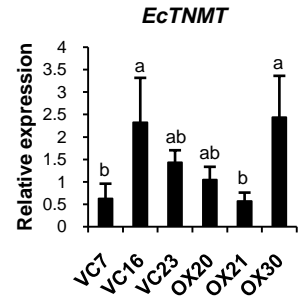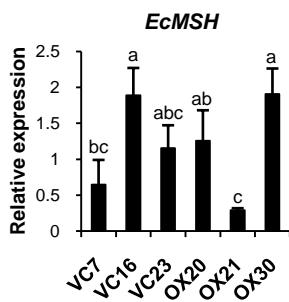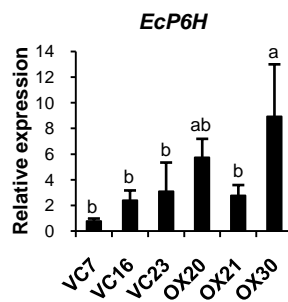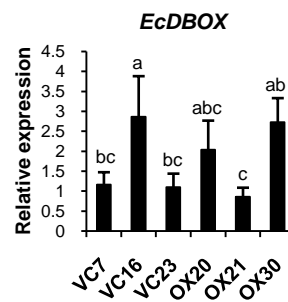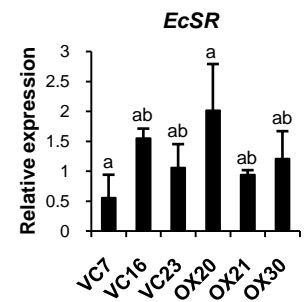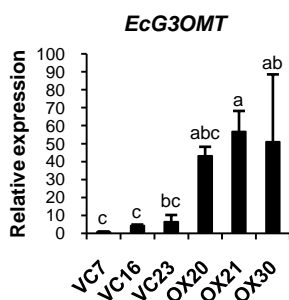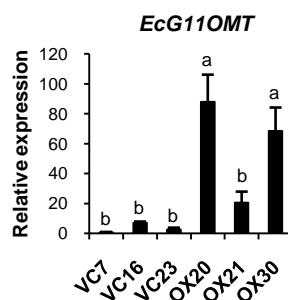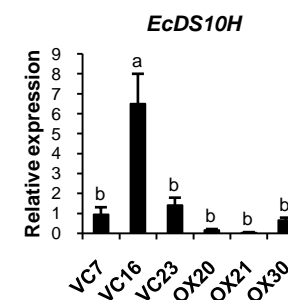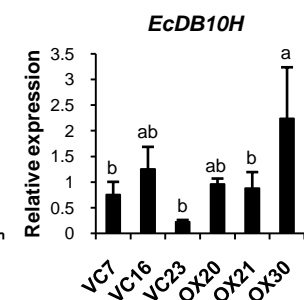

Supplement: S9 Fig — The transcript levels of genes were determined by quantitative RT-PCR. The value is the average of results from three biological replicates. The data are shown as the mean ±s.d.; significance was determined with one-way ANOVA with post-hoc Tukey-Kramer test. (PDF) [file pone.0186953.s009.pdf]

A

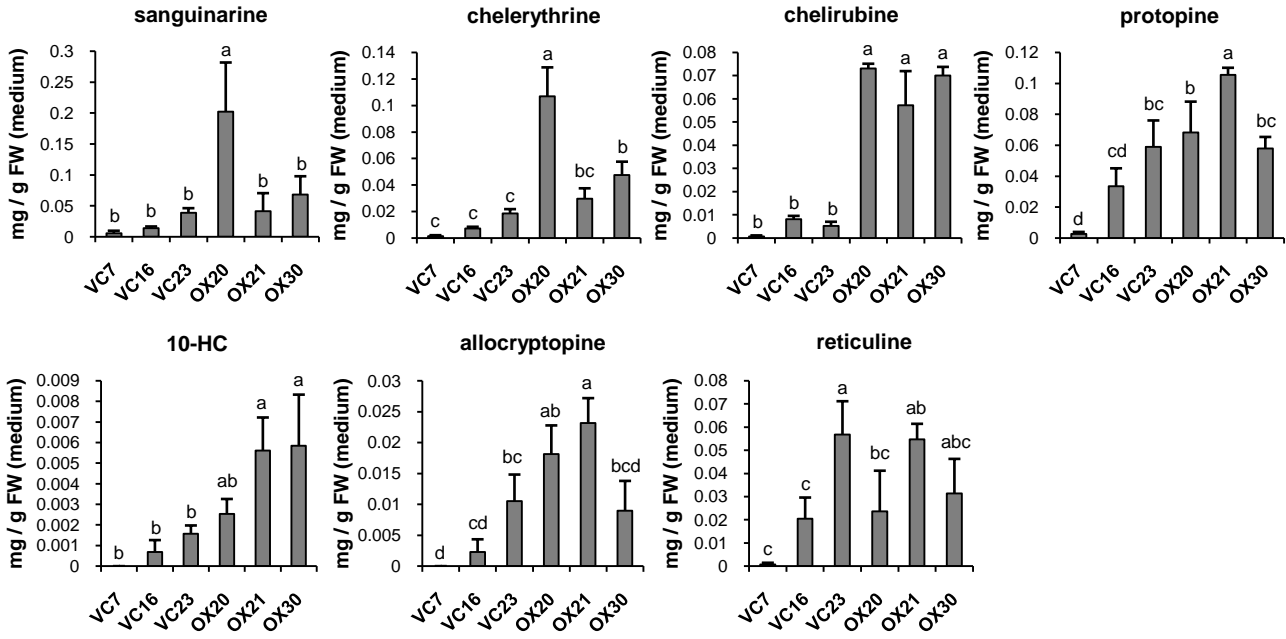

B

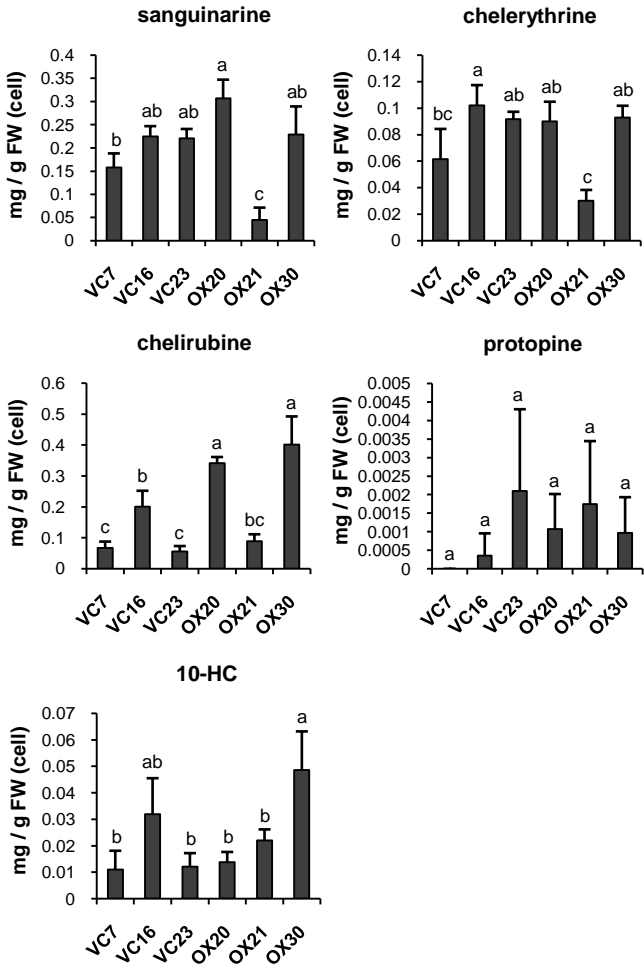

C

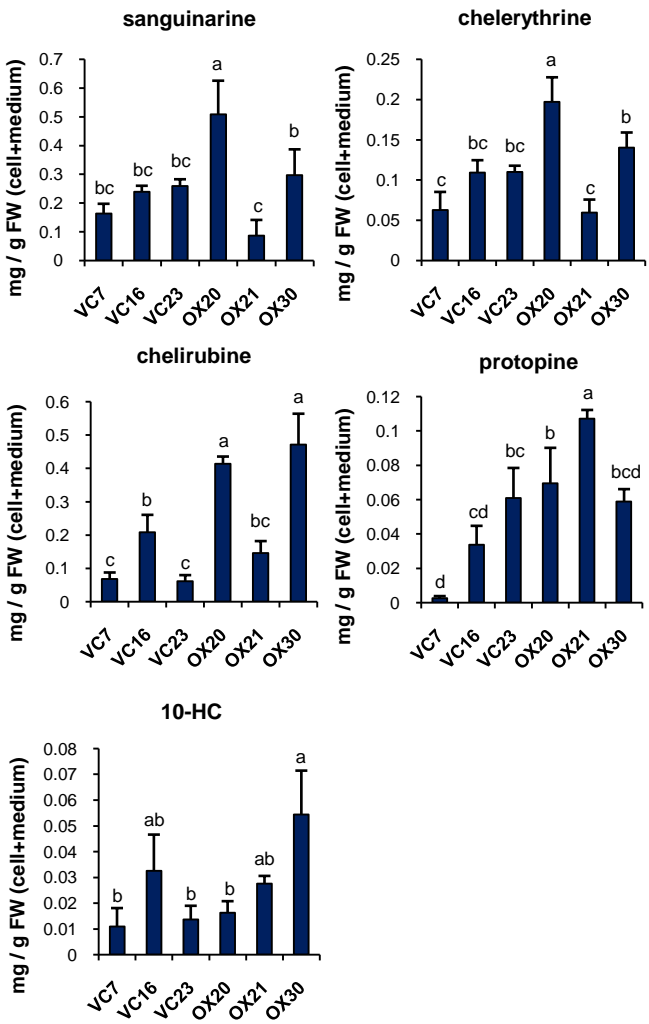

Supplement: S10 Fig — The content of representative alkaloids in the medium (A), cells (B), and both cells and the medium (C) was calculated using the standard curve of sanguinarine. The value is the average of results from three biological replicates. The data are shown as the mean ±s.d.; significance was determined with one-way ANOVA with post-hoc Tukey-Kramer test. (PDF) [file pone.0186953.s010.pdf]

**A**

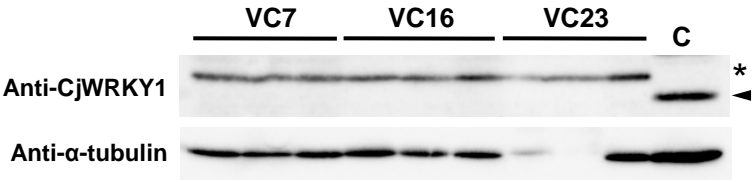

**B**

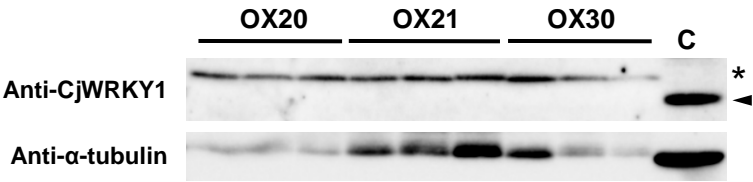

Supplement: S11 Fig — Total protein extracts from VC (A) and CjWRKY1-OX (B) cultured cells were used for immunoblot analysis with anti-CjWRKY1 peptide antibodies and anti-α-tubulin antibody. C: the extract of C. japonica cultured cells served as a positive control. An asterisk and arrow indicate nonspecific protein (possible CjWRKY1 homolog) and CjWRKY1 protein, respectively. (PDF) [file pone.0186953.s011.pdf]

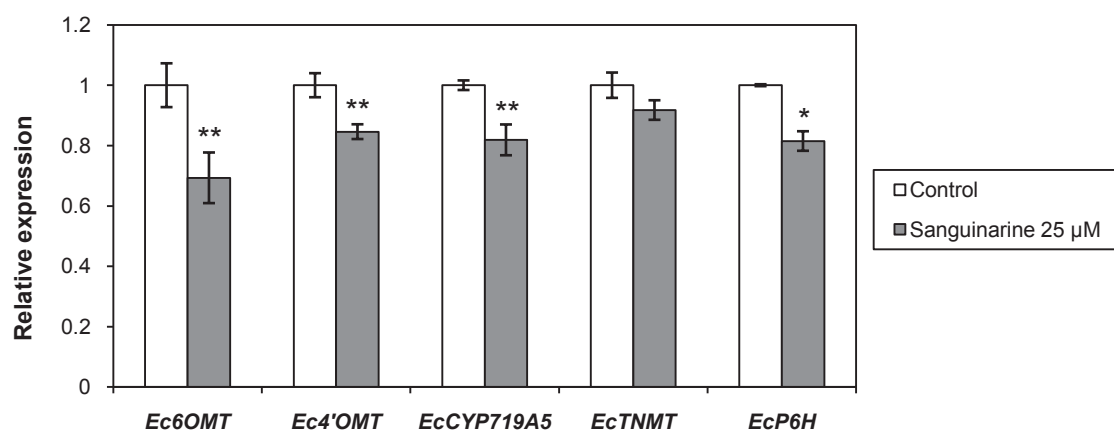

Supplement: S12 Fig — The transcript levels of Ec6OMT, Ec4’OMT, EcCYP719A5, EcTNMT, and EcP6H were determined by quantitative RT-PCR. The relative expression levels were estimated by the standard curve method with three technical replicates and were standardized to the expression of the β-actin gene as the internal control. The average value of the control (0.25% methanol) was set as 1. The data are shown as the mean ±s.d.; *P < 0.05. **P < 0.01, Student’s t-test. (PDF) [file pone.0186953.s012.pdf]
