# Supplementary material for: Modulation of benzylisoquinoline alkaloid biosynthesis by heterologous expression of CjWRKY1 in Eschscholzia californica cells
Source: PLoS One. 2017 Oct 27;12(10):e0186953. doi: 10.1371/journal.pone.0186953 (PMC5659775; doi:10.1371/journal.pone.0186953)
Supplement: S1 Table — (PDF) [file pone.0186953.s013.pdf]

Supporting information

Modulation of benzylisoquinoline alkaloid biosynthesis by heterologous expression of CjWRKY1 in *Eschscholzia californica* cells

Yasuyuki Yamada, Tomoe Shimada, Yukiya Motomura, Fumihiko Sato

Supplementary Table S1. Primers for quantitative RT-PCR of target genes.

Gene		Oligonucleotide sequence (5' to 3')
CjWRKY1	Fw	TGAGCATGCACTCCCTCATA
	Rv	TGGAGGAATATGGGCAAAA
6OMT	Fw	CCTGTTCAACCCGTTGACTTAG
	Rv	CCCAACCCTTAATCAGAAATTTG
CYP80B1	Fw	TCAAACAGTGGTAGGCGAGAGA
	Rv	CAATGGAGTTGGTGGGTGAA
4'OMT	Fw	CCTAGAAGAGGAATCAGAACATCCA
	Rv	TCACTTCTCTCCCTTCCACCA
BBE	Fw	GAGATTAGTAGGAGTTGGGGTGAGA
	Rv	ATTGGAGGGATACTTTGTGGATG
CYP719A5	Fw	CCTGATCTTAGTGAGGATCATTGC
	Rv	ATGCTAGCACTACATGCCATTTCAC
CYP719A2	Fw	GTCGTAATTAATCACTTAACCGTGCTCG
	Rv	GAAAGAAACAGAGCAAATCTTATCCTTTTACC
CYP719A3	Fw	CCTCGTAACTAATATACCAGTGTGGTG
	Rv	GACAACCAAGCAAACTCTTATTCTTGTAC
TNMT	Fw	TTCAGTAGAGGCATGGAGGA
	Rv	TCTTTACTTCCAAGGCCAGG
MSH	Fw	TTCCATCACTTGGTTGGTTAGA
	Rv	CCCAACTCTCAACTACTGAATCAA
P6H	Fw	AACCGTCCTTCCACTAAAGC
	Rv	GGCTCTAACGTCCTTGATGG
DBOX	Fw	AACAAACAGAGCATTCCTCCTC
	Rv	AAAGAAGAATTACGTCGATACGG

SR	Fw	TGAGGAAGTGAAGAACAAAGCA
	Rv	GAACACACCGAGAAACAAAACA
OMT1	Fw	CCTCGTAGTGGATGCTATATTGG
	Rv	GCCTCTTATATGCGGGTATTTCT
CYP1	Fw	AGAACTTCTAGTGAGGTAGCTTTCAC
	Rv	CAAGTATTACGTCTCACTCTAGCTCG
CYP2	Fw	AGATGATGATGAAGCTGGGAAGTTTCAAGAG
	Rv	CTTTTCCTTATAACCTGTTCCAAAATCTAACCACCCTAG
OMT2	Fw	CTGTGGCTCATGTCATTTGTG
	Rv	AATGGATTCGAACATGTCACC
EcbHLH1-1	Fw	CTAGACAGATCGCCTGTTTTGTTG
	Rv	GAGATAGATGACCCTTTAGATAATGAGATCC
EcbHLH1-2	Fw	GGTTGGACAAATCACCAGCTTTAC
	Rv	AAGATAGAGGGGTTCATAATGATAGCAAG
β-actin	Fw	GGTATTGTGCTGGATTCTGGTG
	Rv	GTAGGATTGCGTGGGGTAGTG
